# N^6^-Methyladenosine (m^6^A): A Promising New Molecular Target in Acute Myeloid Leukemia

**DOI:** 10.3389/fonc.2019.00251

**Published:** 2019-04-09

**Authors:** Zaira Ianniello, Alessandro Paiardini, Alessandro Fatica

**Affiliations:** ^1^Department of Biology and Biotechnology “Charles Darwin,” Sapienza University of Rome, Rome, Italy; ^2^Department of Biochemical Sciences “A. Rossi Fanelli,” Sapienza University of Rome, Rome, Italy

**Keywords:** m^6^A, RNA, METTL3, METTL14, AML, leukemia, epitranscriptomics

## Abstract

Recent studies have uncovered an important role for RNA modifications in gene expression regulation, which led to the birth of the epitranscriptomics field. It is now acknowledged that RNA modifiers play a crucial role in the control of differentiation of stem and progenitor cells and that changes in their levels are a relevant feature of different types of cancer. To date, among more than 160 different RNA chemical modifications, the more relevant in cancer biology is the reversible and dynamic N^6^-methylation of adenosine, yielding N^6^-methyladenosine (m^6^A). m^6^A is the more abundant internal modification in mRNA, regulating the expression of the latter at different levels, from maturation to translation. Here, we will describe the emerging role of m^6^A modification in acute myeloid leukemia (AML), which, among first, has demonstrated how mis-regulation of the m6A modifying system can contribute to the development and progression of cancer. Moreover, we will discuss how AML is paving the way to the development of new therapeutic options based on the inhibition of m^6^A deposition.

## Introduction

Chemical modifications in eukaryotic RNAs are known from decades. However, until recent years, their role in cancer development was largely unknown. One of most studied RNA modifications with a well-define role in gene expression regulation is the N^6^-methyladenosine (m^6^A), which is present in all RNA species including mRNAs, lncRNAs, rRNAs, tRNAs, and snRNAs. Here, we will focus on the dynamic m^6^A modification of mRNAs. m^6^A is the most abundant internal modification in mRNA where it can be embedded and erased by specific proteins (1–5). m^6^A mark can specifically recruit reader proteins, such as the YT521-B homology (YTH) domain family of proteins, or it can produce conformational changes within local RNA structures that may indirectly affect the interaction with RNA binding protein (6–9). As a result, m^6^A may regulate mRNA expression at different levels by affecting splicing, nuclear export, stability and translation [reviewed in (10, 11)].

**Figure 1:**
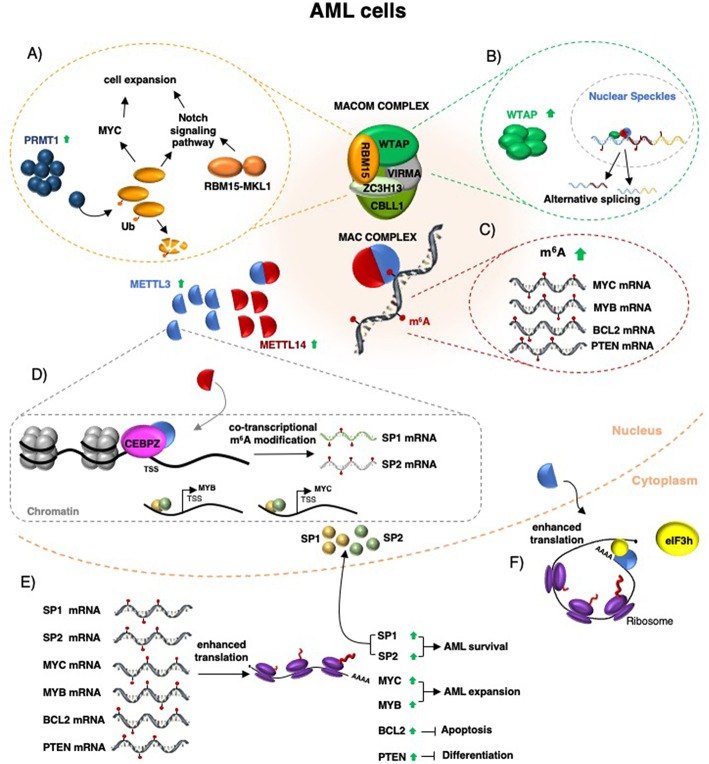
Schematic representation of m^6^A function in AML. The MACOM and MAC complex components are required for AML survival (see main text for details). **(A)** RBM15 protein controls cell expansion and differentiation by regulating c-Myc levels and the Notch signaling pathway. In some forms of pediatric AML, RBM15 is fused to MKL1 and induced leukemia by aberrant regulation of the Notch signaling pathway. **(B)** WTAP protein is upregulated in AML, localized in nuclear speckles, and regulates alternative splicing. **(C)** METTL3 and METTL14 upregulation in AML increases m^6^A methylation of specific mRNAs, including MYC, MYB, PTEN, and BCL2 mRNAs. **(D)** In addition, METTL3 is recruited by CEBPZ on specific promoter regions and this results in co-transcriptional m^6^A methylation of different mRNAs, including that one encoding for the SP1 and SP2 transcription factors. **(E)** Increased m^6^A methylation enhances mRNA translation and produced increased protein levels. **(F)** In AML, METTL3 mis-localizes in the cytoplasm where it can increase the translation of specific mRNAs independently from its catalytic activity by recruiting eIF3h.

The *methyltransferase-like protein 3* (METTL3, also known as MT-A70) and the *methyltransferase-like protein 14* (METTL14) complex (also called MAC, m^6^A-METTL Complex) installs m^6^A in mRNAs and lncRNAs within the DRACH motif (D = A/G/U, R = A/G; H = A/C/U) while the *methyltransferase-like protein 16* (METTL16) is responsible for the m^6^A modification in the U6 snRNA and specific mRNAs and lncRNAs containing the UACAGAGAA sequence within a specific stem-loop structure (4, 5). Notably, METTL3 is the only catalytic component of the MAC but it requires the interaction with METTL14 for RNA binding and m^6^A deposition. The m^6^A modifications present in rRNAs, tRNAs, and U2 and U4 snRNAs are installed by still unknown methyltransferases. The METTL3/METTL14 core complex is assisted by a regulatory complex (named MACOM, m^6^A-METTL-associated complex) composed of *Wilms tumor 1-associated protein* (WTAP), *Vir-like m*^6^*A methyltransferase-associated* (VIRMA, also known as KIAA1429), *Cbl proto-oncogene like 1* (CBLL1, also known as Hakai), *RNA-binding motif 15* (RBM15), and *zinc finger CCCH-type containing 13* (ZC3H13) proteins (12). Interestingly, even if the METTL3/METTL14 consensus sequence can be found along all the mRNA body, m^6^A deposition is enriched nearby the stop codon, 3′-UTR and long internal exons. Therein, it has been suggested that MACOM is responsible for guiding the METTL3/METT14 core complex on a specific region of the mRNA. In view of its reversible nature, m^6^A modification can be removed by ALKBH5 (alkB homolog 5) and FTO (fat-mass and obesity associated protein) proteins. They belong to the AlkB family of the Fe(II) and α-ketoglutarate-dependent dioxygenases, which includes also DNA and histone demethylates (10).

m^6^A was initially identified in pioneering studies in early 1970s in mammals, and later on, in flies, plants, yeast and also RNA viruses (13–16). However, the identification and mapping of m^6^A modification in whole transcriptome in different cell types, states and diseases was possible only in the last years with the development of m^6^A specific antibodies coupled to next generation RNA sequencing technologies (17–19). We are now witnessing the dawn of a new era in cancer biology studies in which gene expression data and epigenetic status of cancer cells are integrated with epitranscriptomics analysis to acquire a better comprehension of the molecular mechanisms that drive tumorigenesis. In this context, the first experimental evidences of a direct involvement of m^6^A deposition in the development of cancer have been obtained in acute myeloid leukemia (AML), a devastating blood cell cancer. In this review, we describe accepted knowledge on the critical role for the m^6^A modifiers, erasers, and readers in AML (Figure 1). Furthermore, in view of the fact that AML represents a remarkable example of malignancy with defects in cell differentiation, we also report recent results obtained in normal hematopoietic stem cells biology. Finally, we discuss the feasibility of chemical inhibition of the writing complex as novel therapeutic option for AML patients.

## m^6^A Deposition is Required for AML Cells Survival

The MACOM components WTAP and RBM15 had already been involved in AML before knowing they were regulators of m^6^A modification. In particular, WTAP was initially identified as interactor of the Wilms' tumor gene [WT1; (20)], whose high expression in AML is associated with poor prognosis (21). Later on, WTAP protein was found upregulated in AML and to act as an oncogene (22). According to its oncogenic role, WTAP knockdown in AML cell lines and AML xenograft model decreased proliferation and induced apoptosis (22). Mechanistically, WTAP silencing in AML cell lines altered alternative splicing (22, 23) and this was in accordance with its previously reported localization in nuclear speckles (24), where the mRNA splicing reaction occurs. Notably, before knowing its role as regulator of m^6^A deposition, the WTAP complex was purified from human cell lines and different components of the MACOM complex were identified as interacting proteins, including VIRMA, RBM15, and CBLL1 (23). Furthermore, in agreement with its role in splicing regulation, it was also demonstrated a transient interaction of the WTAP complex with the splicing machinery (23). Notably, the METTL3 and METTL14 proteins were also identified as WTAP complex interactors in different cell lines (23). However, albeit it was known since 1990s that METTL3 was responsible for m^6^A modification in human mRNAs (25) and that it was implicated in splicing regulation (26, 27), the link between WTAP and m^6^A was initially ignored. Few years later, with the birth of epitransciptomics, the WTAP protein was identified as an important regulator of the METTL3/METTL14 complex (28).

RBM15 is a member of the SPEN (Split-end) family of proteins, a group of proteins with RNA recognition motifs that functions in transcriptional regulation, post-transcriptional processing and nuclear export of mRNAs (29, 30). RBM15 is highly expressed in the hematopoietic system (29). Notably, chromosomal translocation between RBM15 and MKL1 were identified in some forms of pediatrics AML (also referred to as Acute Megakaryoblastic Leukemia). Similar to WTAP, knockdown of RBM15 in AML cell lines inhibited cell differentiation and induced apoptosis (31, 32). In mouse, RBM15 has an important role in regulating HSCs expansion and differentiation. In particular, conditional knockout of RBM15 in the hematopoietic compartment produced a block in B cell differentiation and myeloid and megakaryocytic development (29, 33). Strikingly, it was shown that the effect of RBM15 in the hematopoietic system and AML was partly due to deregulation of the Notch signaling (31, 34) and regulation of c-MYC expression (33), which were later identified as relevant m^6^A targets in haematopoiesis and AML (35–38). In addition, RBM15 protein can be modified by protein arginine methyltransferase 1 (PRMT1), resulting in its ubiquitylation and degradation by proteasome (39). Overexpression of PRMT1 altered alternative splicing and blocks AML cell differentiation (39). Therein, it is very likely that PRMT1 can indirectly control m^6^A deposition by regulating MACOM activity through RBM15 post-translational modification.

More recently, m^6^A modification came into focus of AML studies (40). AML is one of the cancers with the highest expression of both METTL3 and METTL14. Both genes were found upregulated in all subtypes of AML compared to normal hematopoietic cells (37, 38, 41, 42), despite the heterogeneity of this blood cell cancer in terms of chromosomal rearrangement and gene mutations. Moreover, this correlate with higher levels of m^6^A modified mRNAs in AML cell compared to normal hematopoietic progenitors (37, 38, 41). Downregulation of METTL3 and METTL14 expression has been performed in cell lines derived from different human AML subtypes by RNA interference and CRISPR/Cas9 genome editing. In all cases, it has been reported an inhibition of differentiation, proliferation arrest and massive induction of apoptosis (37, 38, 41). Strikingly, the apoptotic response is specific for leukemic cells and it has not been observed in normal hematopoietic progenitors. On the other hand, overexpression of METTL3 and METTL14 promoted AML cell proliferation and the effect was abolished by a catalytic inactive form of METTL3. Despite the common results observed at the cellular level by independent studies, the molecular mechanisms identified differ (Table 1). However, it should be considered that the strong induction of apoptosis observed upon METTL3/METTL14 depletion complicates the subsequent gene expression analysis. Moreover, the techniques to study m^6^A level in the transcriptome are impacted by the methodology, the antibody and sometimes also influenced by culture/experimental conditions (19, 45). In particular, in a first study performed on the MOLM-13 AML cell lines, which carries the *FLT3 internal tandem duplication* (*FLT3-ITD*) that in patients is associated with a more aggressive disease, knockdown of METTL3 resulted in a m^6^A dependent reduction of c-MYC, BCL2 and PTEN mRNA translation while the overexpression of METTL3 produced increased protein levels of all three proteins (37). c-MYC is a well-known oncogene in leukemia, while BCL2 and PTEN are negative regulators of apoptosis and PI3K/AKT pathway, respectively. However, activation of the PI3K/AKT pathway was also observed by increasing the expression of a non-functional METTL3 indicating that it is not merely due to m^6^A modification (37). In addition, the translation defects of BCL2 and c-MYC mRNA are recovered after few days of METTL3 silencing despite the persistence of the proliferation arrest and apoptosis (37). Therein, this indicates that additional mechanisms might be responsible for the observed cellular phenotype.

**Table 1:** The role of m^6^A regulators in leukemia.

**Protein**	**Organism**	**Cell line**	**Feature**	**Molecular mechanism**
METTL3	Human	MOLM-13	FLT3-ITD	METTL3 induces proliferation by methylation of MYB, MYC BCL2 and PTEN mRNAs in order to increase their translation. Knockdown of METTL3 inhibits proliferation, and induce apoptosis (37).
	Human	MOLM-13	FLT3-ITD	METTL3 is recruited on specific TSS by the transcription factor CEBPZ that produces co-transcriptional m^6^A modification and increase translation of SP1 and SP2 transcription factors. Knockdown of METTL3 inhibits proliferation, and induce apoptosis (41).
	Human	K562	BCR-ABL1	METTL3 delocalized in cytoplasm, where it promotes translation of WTAP mRNA (42). Knockdown of METTL3 inhibits proliferation.
	Mouse	Primary AML cells	FLT3-ITD and MLL-AF9	METTL3 is required for AML survival *ex vivo* (41)
METTL14	Human	MONOMAC6 NB4	MLL-AF9 PML-RARα	METTL14 induces proliferation by methylation of MYB, and MYC mRNAs in order to increase their stability and translation. Knockdown of METTL14 inhibits proliferation, and induce apoptosis (38).
	Mouse	Primary AML cells	FLT3-ITD and MLL-AF9	METTL14 is required for AML survival *ex vivo* (41)
METTL16	Mouse	Primary AML cells	FLT3-ITD and MLL-AF9	METTL16 is required for AML survival *ex vivo* (41)
**WTAP**	Human	HL-60 K562	MYC+BCR-ABL1	WTAP knockdown decrease proliferation and increases apoptosis by affecting alternative splicing (22).
RBM15	Mouse Human Human	32DWT18 HEL K562	(Epo)/G-CSFR JAK2 V617F BCR-ABL1	RBM15 expression inhibits myeloid differentiation (31). Knockdown of RBM15 inhibits proliferation, and induce apoptosis (32).
**FTO**	Human	MONOMAC6 MV4-11 NB4	MLL-AF9 KMT2A/AFF1 and FLT3-ITD PML-RARα	Inhibition of FTO activity inhibits AML cell proliferation by regulating ASB2 and RARA mRNA methylation (43, 44).

A second study performed again in MOLM-13 cells, showed that METTL3 is recruited on specific promoter transcription start sites (TSS) by the transcription factor CCAAT enhancer binding protein zeta (CEBPZ, also known as DDIT3 and CHOP) (41). This has been indicated as a mechanism that produces co-transcriptional m^6^A modification on specific RNAs. Notably, CEBPZ gene was found recurrently mutated in different AML subtypes (46, 47), suggesting that this might result in altered recruitment of METTL3. Similarly, METTL14 was also found associated with several TSS but, surprisingly, METTL14 peaks do not overlap with those of METTL3 (41). As METTL14 is strictly required for METTL3 modifying activity, this suggests that the function of the two proteins on chromatin might be independent from m^6^A modification. Interestingly, the *Saccharomyces cerevisiae* METTL14 homologous protein KRF4 was also found associated to chromatin and initially described as a transcription factor (48). Two of the relevant METTL3 modified transcripts identified in MOLM-13 cells were the mRNAs encoding for the SP1 and SP2 transcription factors. SP1 and SP2 belong to the SP/FLF family, whose members have several roles in tumor development (49). Similar to what has been found for c-MYC, upon METTL3 knockdown these transcripts are translated less efficiently even if mRNA levels are not changed. Interestingly, it has also been demonstrated that SP1 and SP2 proteins directly regulate c-MYC transcription (41). Therein, in this case the regulation of c-MYC by METTL3 appears to be indirect. Notably, in MOLM-13 cells silenced for METTL3, overexpression of SP1 rescues cell growth. On the other hand, deletion of SP1 is lethal (41), thus, indicating a relevant role for SP1 in supporting METTL3 function in AML cells. However, SP1 is ubiquitously expressed, and regulates the expression of many genes within the cells. Furthermore, while SP1 knockout mice die early during embryogenesis, mESCs from SP1 knockout animals are viable and they can be induced to differentiate and form embryoid bodies (50). On the other hand, METTL3 knockout in mESCs impairs exit from self-renewal and block differentiation (51), indicating SP1 independent function. It is worth noting that some of the differences between these two studies performed on the same AML cell line might depend from the different antibodies utilized for the identification of m^6^A modified mRNAs. Indeed, it has been shown that the anti-m^6^A antibody influences the efficiency of m^6^A detection (19, 45).

Human AML subtypes are characterized by the presence of gene translocation that results in the expression of oncogenic fusion proteins that contribute to the differentiation block observed in AML (52). Expression of these proteins in normal mouse hematopoietic progenitor cells strongly induced expression of METTL3 and METTL14 (38). More importantly, conditional deletion of METTL14 strongly reduce the oncogenic potential of AML fusion proteins both in primary cells and in recipient mice (38). In addition, the ablation of METTL14 delayed the onset of leukemia and prolonged the survival of mice. Knock-down of METTL14 in the Mono-Mac 6 and NB4 human AML cell lines, which express the oncogenic MLL-AF9 and PML-RARα fusion proteins, respectively, strongly reduced both the mRNA stability and translation of the oncogenes MYB and c-MYC (38). However, it should be considered that the fate of m^6^A modified mRNAs depend on the identity of the reader protein. For instance, in contrast to YTHDF2, IGF2BPs were shown to stabilize mRNAs, including MYC (53). Moreover, ectopic overexpression of MYB and c-MYC partially counteracts the effect of METTL14 depletion on AML cell proliferation and differentiation. Notably, similar to c-MYC, also the MYB promoter is regulated by the SP1 transcription factor (54), whose levels are regulated by m^6^A (see above). Thus, some of the observed phenotypes might be due to indirect effects. Taken together, these results indicate that the oncogenic function of m^6^A writers in AML is mediated by different pathways, which include modulation of SP1, c-MYC, and MYB expression. However, not all the observed cellular phenotypes may be ascribed to the identified regulatory networks and it should be considered that simple m^6^A/target relationships may dictate some phenotypes and complex networks of m^6^A changes within the all transcriptome may underlie others.

Interestingly, a genome-wide CRISPR/Cas9 screening performed in mouse primary leukemia cells expressing both *FLT3-ITD* and *MLL-AF9* fusion genes identified besides METTL3 and METTL14 also METTL16 as critical gene for AML survival (41). METTL16 positively regulates the expression of the human S-adenosylmethionine (SAM) synthetase MAT2A (4, 5, 55), whose expression contribute to appropriate SAM levels. SAM is the major donor of methyl transfer within the cell. Therein, METTL16 expression may indirectly regulate the activity of METTL3/MELL14 and also of many other RNA, DNA and protein methyltransferases.

In some tumors, including AML, METTL3 mis-localize to the cytoplasm where it can promote the translation of specific mRNAs independently from its catalytic domain (42, 56). In particular, it has been shown that METTL3 binds m^6^A modified regions close to the stop codon promoting mRNA circularization and, eventually, mRNA translation by interacting with the eIF3 translation initiation factor subunit eIF3h (57). In AML, higher levels of cytoplasmic METTL3 results in concomitant increase of WTAP protein expression (42). This mechanism might be relevant to increase WTAP protein levels concomitantly to the METTL3/METTL14 complex and sustain its oncogenic role in AML (42). Importantly, the binding of cytoplasmic METTL3 to mRNA occurs independently from METTL14 and it is still not clear how METTL3 would specifically recognize m^6^A mRNAs and, above all, how it will remain stably associated to mRNAs. In the nucleus, the RNA binding activity of METTL3 depends on the presence of a conserved cluster of positively charged residues across the METTL3/METTL14 heterodimer interface and a N-terminal Zinc finger domain in the METTL3 protein (58–61). However, as expected for a writing complex, the affinity of the METTL3/METTL14 heterodimer for RNA is very weak (61). Thus, it is very likely that METTL3 needs specific protein partners for stable mRNA binding in the cytoplasm.

By contrast with the reported oncogenic role of m^6^A in AML, high expression of the FTO demethylase has been also reported in AML carrying the FTL3-ITD, MLL-AF9 or PML-RARA gene translocations (43). Moreover, it was also shown that inhibition of FTO activity in the Monomac6, MV4-11, and NB4 cell lines affected AML cell proliferation capacity (43, 44). These results are in sharp contrast with what has been shown upon METTL3 and METTL14 downregulation in the same AML cell lines (see Table 1). However, in addition to m^6^A, it has been recently reported that FTO also demethylates *N*^6^*, 2-O-dimethyladenosine* (m^6^Am) at the 5' cap in mRNA and *N*^1^*-methyladenosine* (m^1^A) in tRNA (62). Therein, it is very likely that the observed phenotype may be m^6^A independent. The PCIF1 protein (also referred to as CAPAM, cap-specific adenosine methyltransferase) has been recently identified as the methyltransferase responsible for the m^6^Am modification at the 5' cap in mRNA (63, 64). Thus, it would be very interesting to investigate the potential impact of PCIF1 in AML.

## Non-coding Transcripts as m^6^A Targets in AML

Despite many of the studies on m^6^A performed in AML focused on coding RNAs, the METTL3/METTL14 and METTL16 methyltransferases can also modify non-coding transcripts with relevant role in cancer, such as lncRNAs and circular RNAs (circRNA). For example, deletion in the mouse hematopoietic system of the X-inactive specific transcript (Xist), which controls X-dosage compensation in mammals, causes blood cancer (65). Notably, Xist contains several m^6^A modifications that are required for Xist-mediated transcriptional repression (66), thus suggesting that alteration of m^6^A levels might alter Xist function in hematopoietic cells. Metastasis-associated lung adenocarcinoma transcript 1 (MALAT1, also known as NEAT2) is another highly methylated lncRNA (17). MALAT1 is mis-regulated in several human cancers, including leukemia (67). MALAT1 is a nuclear lncRNA that interacts with splicing factors and regulates alternative splicing (68). Moreover, MALAT1 has also been shown to act as a competing endogenous RNA (ceRNA) (69) and as a scaffold for the polycomb repressive complexes 1 and 2 (PRC1 and PRC2) (70–72). Two of the m^6^A marks in MALAT1 affect local RNA structures and regulate the accessibility of RNA binding proteins, a mechanism referred to as m^6^A-riboswitch (73, 74). Therein, it is possible that the higher levels of m^6^A observed in AML might increase the binding of proteins, such as splicing regulators or epigenetic modifiers, that results in gene mis-regulation. Several other lncRNAs have been shown to play critical role in AML (75). Hence, alteration of their structure or expression levels by m^6^A modifications might influence their activity.

Another important class of m^6^A modified molecules is the circRNA family (76). circRNAs are covalently closed circular molecules derived from back-splicing reactions, in which the 5′ splice site of the exon joins with the 3′ splice site of an upstream intron (77). Interestingly, m^6^A modifications in circRNAs differ from the patters of the corresponding linear mRNAs (76) and, more importantly, m^6^A-modified circRNAs regulate the stability of the corresponding linear mRNA in a YTHDF2-dependent manner (76). Thus, it is likely that changes in circRNA m^6^A levels might have a great impact in gene expression regulation. Notably, a correlation between circRNA levels and cell proliferation has already been shown in cancer (78). Moreover, about half of AML patients carry aberrant chromosomal translocations that can produce specific fusion-circRNAs (f-circRNA) between rearranged loci (79). In particular, the PML/RARα translocation, which characterizes a subtype of AML referred to as acute promyelocytic leukemia (APL), produces oncogenic f-circRNAs that have been shown to favor leukemia progression in transgenic mouse models (79). Therein, it will be interesting to study the relationship between the m^6^A modifications and f-circRNA activity. circRNAs may regulate gene expression by several mechanisms, including regulators of splicing and transcription, ceRNAs and protein competitors (77). Moreover, specific circRNA can also be translated in protein by a cap-independent manner (77). This kind of translation is less efficient than cap-dependent translation, but might have an important role under stress condition and in tumor and, importantly, can be regulated by m^6^A modifications (10). A peptide produced from a circular form of the linc-PINT lncRNA has been recently shown to play an important role in glioblastoma tumorigenesis (80) and it is very likely that other examples of translated circRNAs with a role in cancer will follow.

## m^6^A Role in Normal Hematopoiesis

Defects in cell differentiation are a hallmark of AML. In particular, AML is characterized by an accumulation of immature cells which fail to respond to normal regulators of differentiation within the bone marrow (52). Therein, the role of m^6^A RNA modification in normal hematopoiesis has been also analyzed in both purified hematopoietic stem/progenitor cells (HSPCs) and mouse model systems. In cytokine-driven differentiation of human CD34^+^ HSPCs purified from umbilical cord blood, METTL3/METL14 expression decreased with the progression of myeloid differentiation (37, 38). Furthermore, similar to AML cells, knockdown of METTL3 and METTL14 in CD34^+^ HSPCs accelerated myeloid differentiation while their overexpression stimulated proliferation and inhibited differentiation (37, 38). Notably, downregulation of METTL3 and METTL14 in purified HSPCs inhibits cell growth but does not induce massive apoptosis as in AML cells (37, 38). In contrast, conditional knockout of METTL3 in the adult mouse hematopoietic system produced an expansion of the HSCs in bone marrow without any significant alteration in mature myeloid cells production (81). Similar results were obtained with the conditional knockout of the m^6^A reader YTHDF2 (77, 82) but, surprisingly, not upon deletion of METTL14 (81), which is required for METTL3 function. Moreover, HSC with ablation of YTHDF2 have elevated regeneration capacity (77). The differences observed *in vitro* and *in vivo* might reflect the fact that purified cord blood cells differ from their counterparts in the bone marrow (BM). In particular, they have greater proliferative response to cytokines and are less dependent on stromal cells than the corresponding HSCPs in the BM (83). Under physiological conditions, HSPCs homeostasis is maintained by the interaction with stromal cells within the BM and the conditional system utilized for METTL3 and YTHDF2 deletion may also target the stromal cells. Thus, the expansion observed in HSCs *in vivo* might be also due to an alteration of the stem cell niche. However, these data indicate that m^6^A modification plays an important role in maintaining adult HSCs quiescence and, above all, that the inhibition of the m^6^A modification system is well tolerated by the normal HSCs *in vivo*.

## m^6^A as an Anticancer Drug Target

Targeting m^6^A modification writers, erasers and readers by small molecules has been frequently hailed as a potential treatment for several kinds of cancer. Inhibitors targeting 2-oxoglutarate (2OG) and iron-dependent oxygenases [e.g., ALKBH5 (2) and FTO (84)], belonging to the 2OG-dependent nucleic acid oxygenase (NAOX) family and suppressing m^6^A modification demethylation of RNA, have been extensively discussed in a recent review (85). Here, we will focus on therapeutic strategies and small molecules targeting the METTL3/METTL14 complex and discuss their potential applications in cancer treatment.

**Figure 2:**
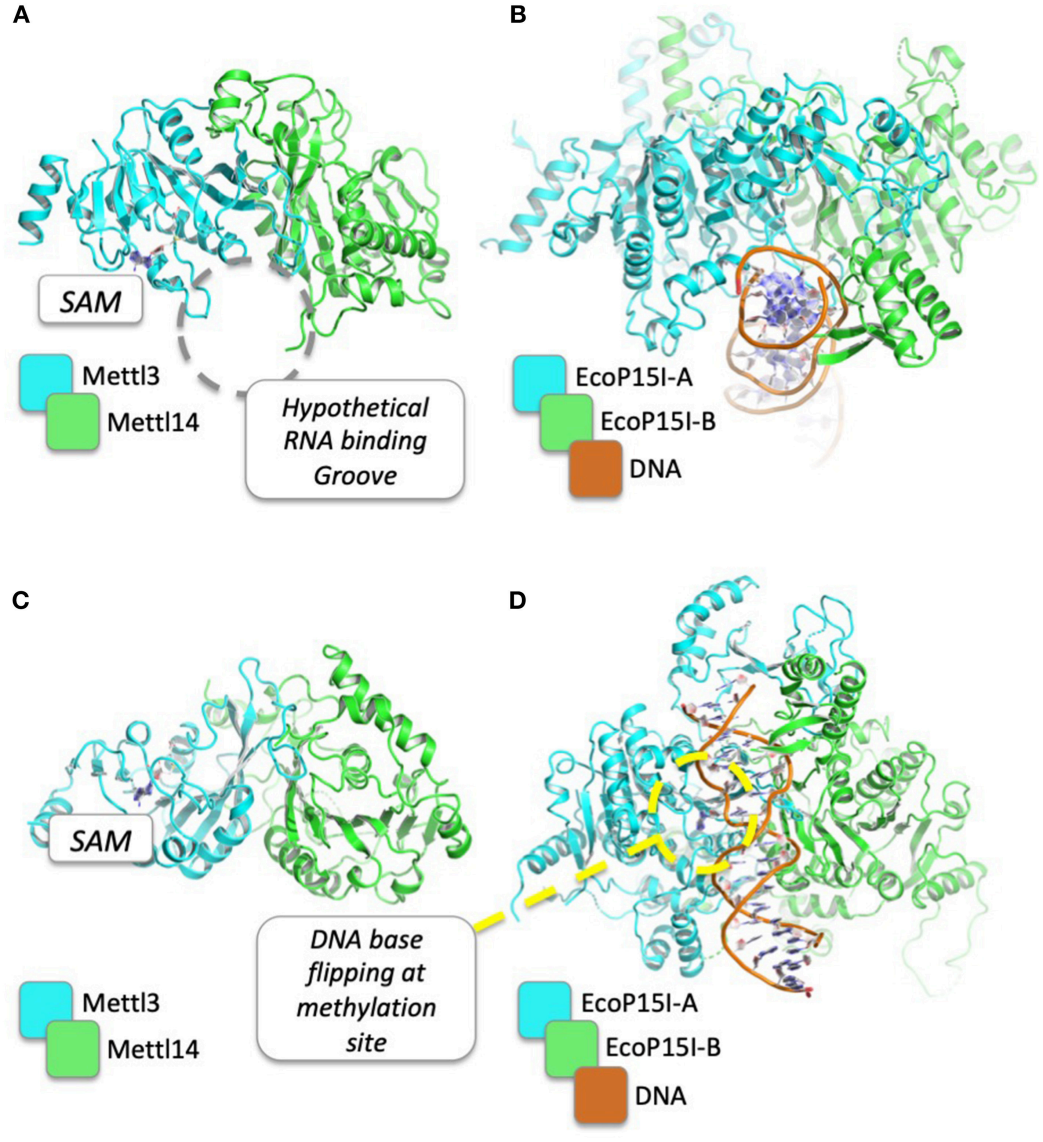
Structural comparison between METTL3/METTL14 complex and EcoP15I in complex with its DNA substrate. PDB codes for METTL3/METTL14 and EcoP15I are 5IL1 and 4ZCF, respectively. The hypothetical binding groove of RNA, just beside the SAM binding site, is shown **(A)** and compared to the DNA binding mode of EcoP15I **(B)**. In **(C,D)**, the structures are rotated by 90°, to show the DNA base flipping that is supposed to be required for DNA/RNA methylation of N6 of adenosine.

Although thus far no inhibitors of METTL3/METTl14 have been reported in the literature, other than the reaction product SAH and the general nucleoside analog Sinefungin (86), the recent availability of high-resolution crystal structures for METTL3/METTL14 complexes [(58–60); (Figure 2)] provides a basis for structure-guided drug design, as the latter can be exploited by computational tools for the rational design of novel inhibitors (87). Current structural information for METTL3 and METTl14 and the potential druggability of these targets are therefore discussed.

The crystal structures of METTL3–METTL14 complex show that both proteins belong to the class I methyltransferase family, the largest homologous group of SAM-dependent methyltransferases. The latter is characterized by a Rossman fold catalytic domain and several conserved sequence motifs (88, 89), and comprises most tRNA, cap, and m6A methyltransferases, as well as DNA methyltransferases (DNMTs), arginine methyltransferases (PRMTs), and some histone-lysine N-methyltransferases (90). METTL3 and METTL14 form an asymmetric heterodimer in which only the former is able to bind SAM and carry out the catalytic methyltransferase reaction. Structural analysis and mutagenesis indicate that both proteins are involved in RNA binding, although a complex with RNA has yet to be determined. However, because METTL3 and METTL14 are both members of the SAM-dependent methyltransferase superfamily, structural insights on RNA substrates binding mechanism can come from the comparisons with the three-dimensional structures between members of the same family. Indeed, as shown in Table 2, METTL3 shows a high structural similarity with other DNA methyltransferases (e.g., Adenine Specific DNA Methyltransferases), for which the dimeric structure in complex with the DNA substrate for methylation is already known [PDB: 1G38; (91)]. On the ground of these homology-based, functional and structural similarities, several conclusions can be drawn on the hypothetical mechanism that is responsible for m^6^A of mRNAs by METTL3/METTL14. Intriguingly, both METTL3 and METTL14 contain sequence motifs characteristic of amino-methyltransferases, including the “DPPW” motif that is equivalent of the “DPPY” sequence in motif IV (92). The linear order of these motifs and structural comparison with other amino-methyltransferases (e.g., EcoP15I, PDB: 4ZCF) suggests that one methyltransferase (i.e., METTL3) plays a more dominant role in adenine methylation, while the other one (i.e., METTL14) plays a more central role in recognition of the surrounding RNA secondary structure (93) (Figure 2). Moreover, a mechanism can be envisaged where, as in the case of other amino-methyltransferases and restriction endonucleases (92), the target adenosine base to be methylated is “flipped” outside the nucleic acid double helix and positioned inside the active site cleft, facing SAM and stabilized with stacking interactions with the Tryptophan residue of the conserved “DPPW” motif (88–90).

**Table 2:** First 10 results of a DALI search in PDB25 for structural similarities among METTL3 homologous proteins.

**PDB**	**Z-Score**	**RMSD**	**Aligned residues**	**Total residues**	**%Identity**	**Description**
5IL0	34.6	0.5	204	211	100	Human Mettl3
5L6D	21.5	2.5	177	238	36	Human Mettl14
1G60	14.4	2.8	167	239	18	Adenine-Specific Methyltransferase Mboiia
5HFJ	14.1	2.9	163	205	18	Adenine Specific Dna Methyltransferase
1NW6	13.3	2.7	169	272	14	Modification Methylase Rsri
5HEK	13.2	2.6	147	177	18	Adenine Specific Dna Methyltransferase
4ZCF	13.1	3.2	182	616	13	Ecop15i Restriction Endonuclease
2ZIF	13.1	3.8	169	244	16	Putative Methylase
1BOO	12.3	2.8	166	283	12	Protein Cytosine-Specific Methyltransferase
5I2H	7.3	2.2	89	335	13	O-Methyltransefrase Family 2

As previously mentioned, in spite of the fact that no inhibitors of METTL3/METTl14 have been found yet, it is reasonable to expect that such a goal is achievable, given that potent and selective inhibitors have been found for the closely related members of the class I methyltransferase family, e.g., protein lysine methyltransferases (PKMTs), protein arginine methyltransferases PRMTs and DNMTs.

**Figure 3:**
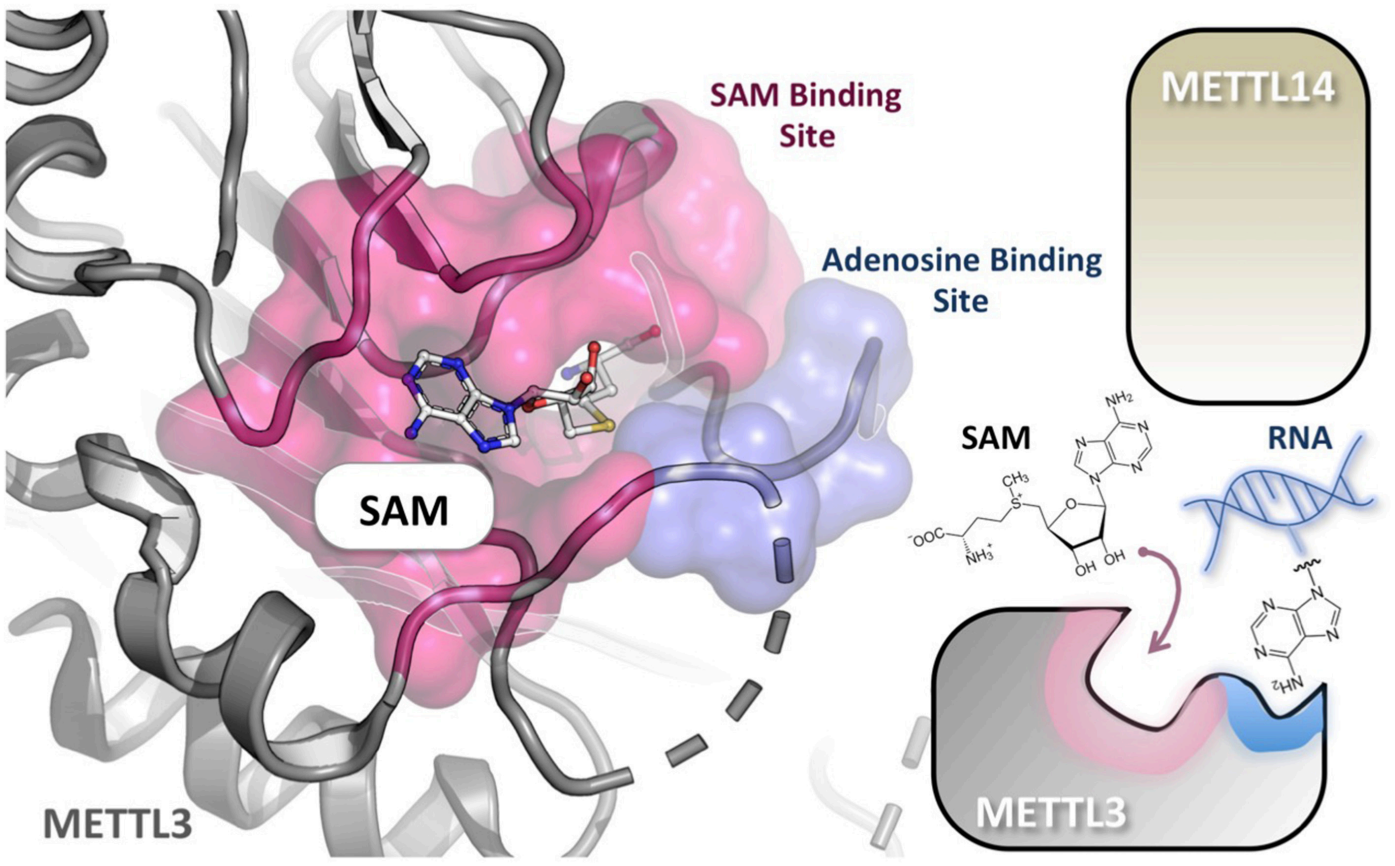
Substrate RNA and cofactor (SAM) of METTL3/METTL14 bind at distinct sites. The crystal structure of the METTL3 monomer in complex with SAM (PDB: 5IL1), shows that the adenosine-binding site (cyan) and the cofactor-binding pocket (purple) are connected by a narrow channel in the protein core. METTL14 is not directly involved in reaction but is probably necessary for RNA binding. A nucleotide base flipping-mechanism is also proposed.

The methyl-donating SAM cofactor and methyl-accepting adenosine substrate bind at distinct sites of METTL3 (71). These binding pockets are common to the Rossmann fold enzymes of class I family and are connected by a narrow channel in the protein core. Therefore, in conceiving potential METTL3/METTL14 inhibitors, nucleosides, and SAM analogs could be considered, or bisubstrates ligand mimicking both (Figure 3). For example, Azacytidine (Vidaza) and Decitabine (Dacogen) are nucleoside analogs targeting DNMTs and being approved for clinical use in hematological malignancies (94). Unfortunately, these drugs display poor bioavailability and toxicity. By contrast, small-molecule inhibitors binding within the SAM pocket have shown good pharmacological properties and oral bioavailability and are currently under clinical investigation as cancer therapeutics mainly against PKMT and PRMT protein families (95–97). Despite the fact that these SAM mimicking inhibitors share the same cofactor-binding site, side chains lining the SAM-binding cleft are usually not conserved. As in the case of protein kinases, therefore, such structural diversity could be exploited, at least in principle, to achieve highly selective inhibition (98). For example, the methylthioadenosine endogenous compound is a highly specific PRMT5 inhibitor (99). However, structure-based design of bisubstrate inhibitors holds great promise for far higher selectivity, compared to SAM and nucleoside analogs. Recently, (100) designed DNMT3A and DNMT1 bisubstrate inhibitors by linking together SAM and the deoxycytidine substrate. This approach resulted in quinazoline–quinoline derivatives as potent inhibitors, some showing also isoform selectivity. The most potent inhibitors induced demethylation of CDKN2A promoter in colon carcinoma HCT116 cells and its reactivation after 7 days of treatment. In this study, the authors highlighted the importance of the nature and rigidity of the linker between the two moieties for optimal inhibition, an issue that should be taken into account also in designing potential bisubstrate inhibitors of METTL3/METTL14.

In conclusion, the high structural diversity of these compounds pinpoints the significant range of inhibition strategies that can be conceived to target the class I methyltransferases. Although the structural details of the various members of this family are unique, the success stories of drug design for several enzymes belonging to this family portends the likely achievement of discovering potent and selective inhibitors of METTL3/METTL14.

## Author Contributions

AF and AP wrote the manuscript. ZI revised and edited manuscript and prepared figures.

### Conflict of Interest Statement

The authors declare that the research was conducted in the absence of any commercial or financial relationships that could be construed as a potential conflict of interest.
